# Comparison of Major Clinical Outcomes between Accredited and Nonaccredited Hospitals for Inpatient Care of Acute Myocardial Infarction

**DOI:** 10.3390/ijerph18063019

**Published:** 2021-03-15

**Authors:** Bo Yeon Lee, You Jin Chun, Yo Han Lee

**Affiliations:** 1 Health Insurance Review and Assessment Service, Wonju 26465, Korea; tarasyn99@korea.ac.kr; 2 Korea Institute for Healthcare Accreditation, Seoul 07238, Korea; cyj@koiha.or.kr; 3 Graduate School of Public Health, Ajou University, Suwon 16499, Korea

**Keywords:** hospital accreditation, acute myocardial infarction, clinical outcomes

## Abstract

Hospital accreditation programs are used worldwide to improve the quality of care and improve patient safety. It is of great help in improving the structure of hospitals, but there are mixed research results on improving the clinical outcome of patients. The purpose of this study was to compare the levels of core clinical outcome indicators after receiving inpatient services from accredited and nonaccredited hospitals in patients with acute myocardial infarction (AMI). For all patients with AMI admitted to general hospitals in Korea from 2010 to 2017, their 30-day mortality and readmissions and length of stay were compared according to accreditation status. In addition, through a multivariate model that controls various patients’ and hospitals’ factors, the differences in those indicators were analyzed more accurately. The 30-day mortality of patients admitted to accredited hospitals was statistically significantly lower than that of patients admitted to nonaccredited hospitals. However, for 30-day readmission and length of stay, accreditation did not appear to yield more desirable results. This study shows that when evaluating the clinical impact of hospital accreditation programs, not only the mortality but also various clinical indicators need to be included, and a more comprehensive review is needed.

## 1. Introduction

Hospital accreditation programs are being introduced to improve the quality of care and ensure patient safety in many countries around the world [1,2,3]. Although the criteria for accreditation and elements of evaluation vary according to each country and region, it is gradually developing from a structural evaluation-oriented accreditation to a direction that encompasses both the care process and the clinical outcomes resulting from it, and the latter is more emphasized in recent years [3,4,5,6]. As a result, there is a growing interest and research on whether this hospital accreditation program improves patients’ clinical outcomes.

The results of previous studies on the clinical impact of hospital accreditation programs are mixed and varied in a short summary. This is because each study used (i) different diseases of interest, (ii) different clinical indicators, and (iii) different methods of comparison, making it difficult for studies to produce consistent results [7,8,9,10,11,12]. This shows that hospital accreditation can have different impacts depending on the disease, clinical indicators, and methods of analysis. Therefore, the use of the most common and representative disease, clinical outcome indicators, and comparative analysis methods are helpful in assessing the clinical impact of accreditation programs by intuitively approaching this complex problem.

Acute myocardial infarction (AMI) is acute, severe, and has a relatively high prevalence, so it is relatively easy to measure the outcome or performance of medical services for it in a population, and it is used as an indicator reflecting overall medical process outcomes [13,14]. Among the numerous clinical indicators, 30-day mortality and readmission and length of stay are expected to be more directly related to quality of care, and they are widely used clinical quality indicators worldwide [15,16].

South Korea’s Ministry of Health and Welfare and The Korea Institute for Healthcare Accreditation (KOIHA), the organization in charge of the evaluation process has been implementing hospital accreditation programs since 2004 [17], but there are still few studies on the clinical impact of this state-led program targeting the Korean population [18]. We obtained clinical data, including all AMI patients admitted to all general hospitals in Korea, by taking advantage of the single health insurance system in South Korea (hereafter Korea). The purpose of this study was to evaluate whether the key clinical outcome indicators, 30-day mortality and readmission, and length of stay of AMI patients admitted to accredited and nonaccredited hospitals were indeed different between the two groups.

## 2. Materials and Methods

### 2.1. Study Population and Data Sources

This study included all patients admitted to a general hospital for AMI (I21) based on the International Classification of Diseases (ICD)-10 from 1 January 2010 to 31 December 2017 in Korea. General hospitals in Korea refers to hospitals with more than 100 beds and at least 7 specialties, all of which are training hospitals, with a total of 352 as of 2018 (Supplementary Table S1). Patients who were admitted to the hospital for AMI during the period when hospital accreditation effectively applied were classified as patients in accredited hospitals (*n* = 183), whereas those who did not were classified as patients in nonaccredited hospitals (*n* = 169). Clinical information on these patients was obtained from the claims data of the National Health Insurance Corporation, a single-payer system covering the whole population [19]. Data on the deaths of these patients were obtained from the National Statistical Office of Korea. Information on hospital accreditation status and application period was obtained from KOIHA’s official website. The protocol of this study was approved by the Institutional Review Board of Korea University (KUIRB-2018-0095-01).

### 2.2. Variables and Risk Adjustment

As covariate variables for constructing a multivariate model, both patient and hospital variables were extracted from health insurance claims data. Patient variables used were age, gender, type of health coverage (health insurance versus medical aid), level of comorbidity, and hospitalization route (through emergency room versus outpatient care). Hospital variables include the number of medical workers, hospital ownership, and the region where the hospital was located. The number of doctors and nurses was expressed as the number per 100 beds. Hospital ownership was classified into public, corporate and private, and hospital locations were classified into metropolitan and nonmetropolitan regions.

The 30-day mortality and readmission and length of stay were used as outcome indicators. The 30-day mortality refers to cases of death within 30 days of being hospitalized after being diagnosed with AMI for the first time since 2010. The 30-day readmission refers to cases of rehospitalization under the AMI code within 30 days after discharge. Length of stay refers to the number of days from the start of hospitalization to discharge or death.

Since the severity and clinical risk of patients admitted to accredited and nonaccredited hospitals were expected to be different, we classified each patient’s risk using the Charlson Comorbidity Index (CCI) and put this information into a multivariate model mentioned later. The CCI is a method of categorizing comorbidities of patients based on the ICD diagnosis codes found in administrative data. Each comorbidity category has an associated weight (from 1 to 6), based on the adjusted risk of mortality or resource use, and the sum of all the weights results in a single comorbidity score for a patient. A score of zero indicates that no comorbidities were found. The higher the score, the more likely the predicted outcome will result in mortality or higher resource use [20,21,22]. CCI was classified as 0 if the CCI score was 0, 1 if it was 1–2, and 2 if it was 3 or more. We reviewed the list of diseases for calculating the CCI applied to the Korean population and selected the 14 most commonly used diseases, as shown in Table 1 [23].

### 2.3. Statistical Analyses

First, the absolute values of the three clinical indicators of the two groups of patients admitted to hospitals that are accredited and those that are not were presented. Both the 30-day mortality and readmissions were presented as N and % and length of stay as an average ± standard deviation. Second, the multiple logistic regression analysis was performed to determine whether the 30-day mortality and readmission rates were related to accreditation status. All patient and hospital factors presented in Table 2 were introduced into the model as covariates. The model equation was as follows.

$$\begin{array}{c} \log\frac{Y}{1-Y}\;(Y=\mathrm{dead}\;\mathrm{within}\;30\;\mathrm{days}\;\mathrm{or}\;\mathrm{readmit})\;=\beta 0\left(intercept\right)+\beta 1\cdot sex+\beta 2\cdot age+\\ \beta 3\cdot insurance\;type+\beta 4\cdot admission\;type+\beta 5\cdot health\;workforce+\\ \beta 6\cdot ownership+\beta 7\cdot region+\beta 8\cdot accreditation\;status\;(Y/N) \end{array}$$

Third, the multiple linear regression analysis was performed to determine whether the length of stay was related to accreditation status. The model equation was as follows.

$$\begin{array}{c} Y\;(\mathrm{length}\;\mathrm{of}\;\mathrm{stay})=\beta 0\left(intercept\right)+\beta 1\cdot sex+\beta 2\cdot age+\beta 3\cdot insurance\;type+\\ \beta 4\cdot admission\;type+\beta 5\cdot health\;workforce+\beta 6\cdot ownership+\beta 7\cdot region+\\ \beta 8\cdot accreditation\;status\;(Y/N)\;\in \left(error\;term\right) \end{array}$$

For data processing and all statistical analyses, SAS 9.4 was used (version 9.3, SAS Institute Inc., Cary, NC, USA).

## 3. Results

The total number of subjects for this study, that is, the number of patients admitted to general hospitals in Korea for AMI between 2010 and 2017, was 80,262. Of these, 67,939 patients were admitted to accredited hospitals, while 12,323 patients were admitted to nonaccredited hospitals (Table 2). There were no significant differences in sex, age, and type of health insurance between the two patient groups, but patients admitted to accredited hospitals were more often admitted through emergency rooms than nonaccredited hospitals. The characteristics of the hospitals in which the two groups were admitted were significantly different compared to the difference in patient factors. Compared to nonaccredited hospitals, accredited hospitals tend to be located in the metropolitan area, have more beds, have less private ownership, and have more medical personnel (Supplementary Table S1).

Table 3 shows the results of a multivariate model that controls several patient and hospital factors along with the direct differences between the two groups of the three outcome indicators. The 30-day mortality rate of patients admitted to accredited hospitals was about half of that of those admitted to nonaccredited hospitals (1.51% vs. 3.29%). The adjusted odds ratio of the 30-day mortality between the two groups was 0.845, indicating that the mortality rate of the accredited hospital was statistically significantly lower than that of the nonaccredited hospital.

About 10% of patients with AMI admitted to accredited hospitals were readmitted within 30 days after discharge, whereas less than 5% of those admitted to nonaccredited hospitals. However, this difference was canceled out in the multivariate model, and there was no statistically significant difference in readmission rates between the two groups.

The length of stay among patients admitted to accredited hospitals and nonaccredited hospitals was 8.59 and 10.49 days, respectively, but multivariate analysis showed that patients in accredited hospitals had longer hospital stays.

## 4. Discussion

### 4.1. Main Findings and Their Implications

In the present context where there is surprisingly insufficient empirical evidence for hospital accreditation programs that are expected to have a positive effect on the care process and patient outcome, this study investigated whether hospital accreditation had a beneficial effect on the patient’s clinical outcome using the most commonly addressed disease, the most commonly used outcome indicators, and the most intuitive research method (accredited vs. nonaccredited). AMI is well suited for evaluation of accreditation impact on the clinical outcome as it is a common diagnosis and major burden of disease for which quality measures have been established [5]. Measuring and reporting 30-day mortality for AMI is a widely used way of hospital performance evaluation across countries. In some countries, 30-day mortality due to AMI must be reported annually for all hospitals [13,24,25]

The key findings of this study were that AMI patients admitted to accredited hospitals were somewhat less likely to die within 30 days of admission compared to patients admitted to nonaccredited hospitals. However, in other important outcome indicators, readmission rate and length of stay, no positive effects of accreditation were observed. Patients admitted to accredited hospitals were expected to have a higher absolute risk of death because their CCI and rate of admission via emergency room was higher than those admitted to nonaccredited hospitals, but the short-term mortality index of this study was rather opposite, which shows that hospital inpatient services can make an important difference. In other words, it can be said that hospital factors that can offset or compensate for patient factors are possible.

As shown in Supplementary Table S1, the number of manpower and beds in accredited hospitals is relatively large, and because these hospitals are highly likely to be located in a metropolitan area, the quality of the care functions of these hospitals might be superior to that of nonaccredited hospitals. In addition, it needs to be emphasized that a higher proportion of nonaccredited hospitals are privately operated and hold relatively fewer inpatients than accredited hospitals. These disadvantageous characteristics of nonaccredited hospitals may have caused worse AMI care compared to those of accredited hospitals. We tried to minimize the influence of hospital factors to some extent by including some of these hospital-specific characteristics, such as personnel level, owner, and region variables in the multivariate regression model, but this alone does not sufficiently adjust the disadvantages of nonaccredited hospitals. It needs to be emphasized that the low mortality rate within 30 days at the accredited hospital can be caused by the hospital characteristics apart from the effect of the accreditation itself. However, the fact that the results of the study on readmission rate and length of stay are not drawn in the same direction as the mortality rate within 30 days shows that the impact of the clinical outcome of accreditation should still be carefully measured and evaluated. Since this study covers all general hospitals in Korea, our findings might be generalized to the whole population and could be utilized for the operation of hospital accreditation programs in Korea.

South Korea currently conducts hospital accreditation through KOIHA, which comprehensively evaluates the rights and safety of patients for all hospitals, activities to improve the quality of medical services, progress and performance of medical services, personnel management, and patient satisfaction [26]. The evaluation criteria for this program consist of four areas: Basic Values, Patient Care, Administration, and Performance Management. Among these, the areas of basic value, administration, and performance management consisted of 44 indicators covering the structure and administrative aspects of the hospital. The patient care area, a key component of accreditation, consists of 47 indicators that measure the patient care process [17].

### 4.2. Comparison with Other Studies

Much research has been conducted on the impact of medical institution accreditation on the clinical outcomes of patients. Among them, a couple of studies that compare patient outcomes in accredited hospitals and nonaccredited hospitals, that is, studies with similar research methods to ours, were found. The main findings of these studies are a mix of positive and negative effects of accreditation on clinical outcomes. In particular, for short-term mortality indicators, accreditation has a somewhat positive effect, but for indicators that are not so dramatic such as length of stay, hospital accreditation programs have not been shown to have a clear effect.

In a study involving public hospitals in Denmark, conducted in the most similar manner to this study, a regression analysis was performed after adjusting patient characteristics, CCI, and hospital characteristics. The 30-day mortality rate was significantly lower in the patient group admitted to the high-compliant hospital to accreditation compared to the patient group admitted to the low-compliant hospital, but the risk of acute readmission was not different between the two groups [27]. Both the study method and results were similar to those of this study.

In another study comparing the difference in the average length of stay and acute readmission between patient groups admitted to fully accredited and partially accredited hospitals, the average length of stay in accredited and nonaccredited hospitals was 4.51 days (95% CI: there was no difference between the two groups at 4.46–4.57) and 4.54 days (95% CI: 4.50–4.57), respectively, and acute readmission rates were 13.70% (95% CI: 13.45–13.95) and 12.72% (95% CI: 12.57–12.86), respectively, so there was no difference between the two groups [28].

In a study that evaluated the association between accreditation and mortality within 30-day mortality in 80 diseases, which are the leading causes of death within 30 days of hospitalization, the 30-day mortality rate of accredited hospitals was 4.14% (95% CI: 4.00–4.28) and partially accredited hospitals was 4.28% (95% CI: 4.20–4.37), which showed no difference between the two groups, but after adjusting the patient and hospital factors, the adjusted OR was 0.83 (95% CI: 0.72–0.96), which was significantly lower in the certified hospitals [29].

From the results of the previous studies above, it can be seen that the hospital accreditation program tends to reduce short-term deaths from acute and severe diseases such as AMI but has no significant effect on other indicators, and these results are consistent with those of our study.

### 4.3. Future Directions for Research

Above all, it is necessary to fully admit that evaluating the clinical impact of hospital accreditation is very complex, and no premature conclusions should be drawn. As revealed in our and existing studies, contradicting results may be derived depending on the disease and indicators used. Therefore, it is necessary to include as many diseases and indicators in the study as possible so that the partial results of each study can be compared with each other.

Research methods can be broadly divided into two types: one that measures before and after accreditation clockwise and a method that compares according to accreditation status, as in this study. Since both methods have advantages and disadvantages, future research needs to be conducted after sufficient consideration of the methodology.

Lastly, in order to maximize the comparability of research results, the basic knowledge of the accreditation system in each country should be shared to some extent with the academic world, and it is necessary to provide sufficient information on the content and format of accreditation.

### 4.4. Limitation of the Study

Our study has several limitations. First, outcome indicators can measure the direct impact of health care, can be used as a long-term measurement framework, and are advantageous when measuring overall system performance. However, the risk adjustment must be made and the difficulty of measuring is a limitation of the outcome indicator. Although the complete level of risk adjustment was not possible in this study, a certain degree of risk adjustment was achieved through the two methods used in previous studies, namely CCI calculation and the use of a multivariate model that controls various confounding variables. Although not taken into account in this study, it is also important to distinguish whether AMI is ST-elevation MI (STEMI) or non-ST-elevation MI (NSTEMI) in risk adjustment, because the mortality rate within 30 days of STEMI is only half that of NSTEMI [30].

Second, since the results of this study and the achievements of the certification items were not linked, it was not possible to provide a wealth of possible explanations for the various results of the study. In future studies, if possible, a more detailed and practical analysis is needed by grasping the relationship between the result of measuring the impact of each accreditation and the corresponding accreditation components.

## 5. Conclusions

This study was conducted to compare the levels of core clinical outcome indicators after receiving inpatient services from accredited and nonaccredited hospitals in patients with AMI. The 30-day mortality of patients admitted to accredited hospitals was statistically significantly lower than that of patients admitted to nonaccredited hospitals. However, for 30-day readmission and length of stay, accreditation did not appear to yield more desirable results. This study shows that when evaluating the clinical impact of hospital accreditation programs, not only the mortality but also various clinical indicators need to be included, and a more comprehensive review is needed.

## Figures and Tables

**Table 1:** Disease list used for calculating the Charlson Comorbidity Index.

Disease	Diagnostic Codes Compatible with the ICD-10 Coding	Charlson Comorbidity Index Score
**Diabetes mellitus**	E10–E14	1
**Congestive heart failure**	I50	1
**Peripheral vascular disease**	I70–I79	1
**Cerebrovascular disease**	I60–I69	1
**Dementia**	F03, G30	1
**Chronic pulmonary disease**	J41–J45, J47, J64	1
**Rheumatic or connective tissue disease**	M30–M36, M06	1
**Gastric or peptic ulcer**	K25, K26	1
**Mild liver disease**	B18, B19, K70–K77	1
**Hemiplegia or paraplegia**	G80–G81	2
**Moderate or severe renal disease**	N17–N19	2
**Any malignancy, including lymphoma and leukemia**	C81–C96	2
**Metastatic solid tumor**	C76–C80	6
**Acquired immune deficiency syndrome**	B20–B24	6

**Table 2:** General characteristics of study subjects according to hospital accreditation status.

		Patients Admitted to Accredited Hopitals *n* = 67,939 (%)		Patients Admitted Nonaccredited Hospitals *n* = 12,323 (%)	
**Patient**					
Sex	Male	53,293	(78.44)	8511	(69.07)
	Female	14,646	(21.56)	3812	(30.93)
Age	<50	7706	(11.34)	1620	(13.15)
	50–64	27,546	(40.55)	4508	(36.58)
	65+	32,687	(48.11)	6195	(50.27)
Insurance Type	Medical aid	4916	(7.24)	1217	(9.88)
	Insurance	63,023	(92.76)	11,106	(90.12)
Charlson comorbidity index	0	55,419	(81.57)	10,556	(85.66)
	1	11,811	(17.38)	1662	(13.49)
	2	709	(1.04)	105	(0.85)
Admission Type	Via emergency room	45,859	(67.50)	5192	(42.13)
	Via outpatient care	22,080	(32.50)	7131	(57.87)
**Hospital**					
Workforce per 100 beds	No. of physician	31.70		15.28	
	No. of nurses	87.97		52.59	
Ownership	Public	6692	(9.85)	67	(0.54)
	Corporate	56,759	(83.54)	8698	(70.58)
	Private	4488	(6.61)	3558	(28.87)
Region	Metropolitan	32,539	(47.89)	3329	(27.01)
	Nonmetropolitan	35,400	(52.11)	8994	(72.99)

Note: For all patient and hospital variables, the *p*-values for the difference between the two groups were <0.001. Brackets: percentages; public ownership: hospitals owned by central or local governments; corporate ownership: hospitals owned by nonprofit organizations; individual ownership: hospital owned by an individual.

**Table 3:** Comparisons of three clinical outcomes between accredited and nonaccredited hospitals with multivariate modeling.

Clinical Outcomes	Patients Admitted to Accredited Hopitals *n* = 67,939 (%)	Patients Admitted Nonaccredited Hospitals *n* = 12,323 (%)	Adjusted OR	(95% CI)
**30-day mortality (*n*, %)**	1029 (1.51)	406 (3.29)	0.845	(0.777–0.929)
**30-day readmission (*n*, %)**	6554 (9.67)	550 (4.46)	1.08	(0.973–1.200)
**Length of stay** **(Mean ± SD)**			**Adjusted β (*p*-value)**	
	8.59 ± 1.62	10.49 ± 1.84	0.292 (<0.0001)	

Note: adjusted for sex, age, insurance type, comorbidity, admission type, health workforce, and hospital ownership and region. Multiple logistic and linear regression were used for 30-day mortality and readmission and length of stay, respectively. Nonaccredited hospitals were set as the reference.

## Supplementary Materials

The following are available online at https://www.mdpi.com/1660-4601/18/6/3019/s1, Table S1: Comparisons of hospital characteristics according to accreditation status.
