# Supplementary material for: Comparison of Major Clinical Outcomes between Accredited and Nonaccredited Hospitals for Inpatient Care of Acute Myocardial Infarction
Source: Int J Environ Res Public Health. 2021 Mar 15;18(6):3019. doi: 10.3390/ijerph18063019 (PMC8001555; doi:10.3390/ijerph18063019)
Supplement: Supplementary file 1 [file ijerph-18-03019-s001.pdf]

Supplementary Table S1. Comparisons of hospital characteristics according to accreditation status.

Hospital Characteristics		Accredited Hospitals (*n* = 183)		Nonaccredited Hospitals (*n* = 169)		*p*
		n	%	n	%	
Location	Metropolitan	77	44.51	50	29.59	0.0010
	Chungchung	19	10.98	15	8.88	
	Kyungsang	51	29.48	53	31.36	
	Jeolla	17	9.83	44	26.04	
	Kangwon	9	5.20	7	4.14	
No. of beds	Less than 200	24	13.87	65	38.46	<0.0001
	201–300	51	29.48	76	44.97	
	301–400	32	18.50	16	9.47	
	More than 400	66	38.15	12	7.10	
Ownership	Public	41	23.70	4	2.37	<0.0001
	Corporate	110	63.58	100	59.17	
	Private	22	12.72	65	38.46	
No. of medical personnel	Doctors	90.4		27.3		<0.0001
	Nurses	216.3		90.5		<0.0001

Note: Chi-square test or *t*-test were used for categorical and continuous data, respectively.
